# Perspective: How Fast Dynamics Affect Slow Function in Protein Machines

**DOI:** 10.1021/acs.jpcb.3c00705

**Published:** 2023-05-17

**Authors:** Gilad Haran, Inbal Riven

**Affiliations:** Department of Chemical and Biological Physics, Weizmann Institute of Science, Rehovot 7610001, Israel

## Abstract

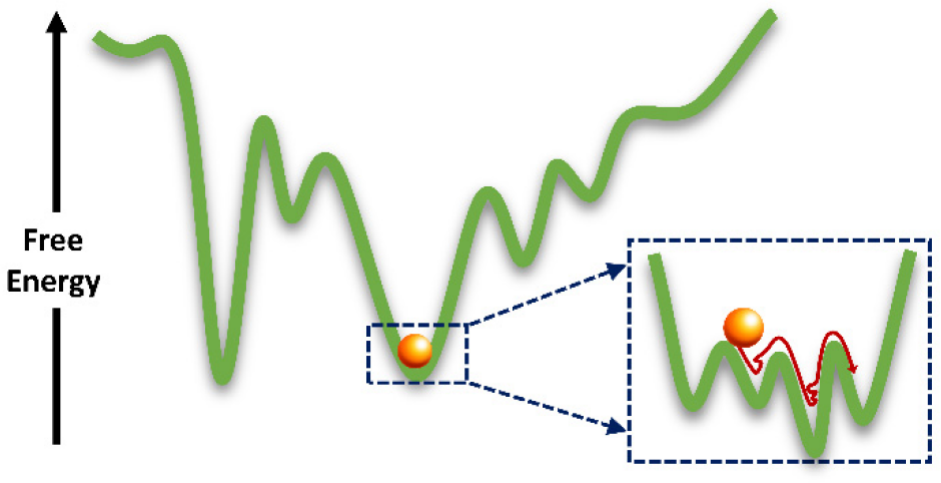

Internal motions in proteins take place on a broad range of time- and space-scales. The potential roles of these dynamics in the biochemical functions of proteins have intrigued biophysicists for many years, and multiple mechanisms to couple motions to function have been proposed. Some of these mechanisms have relied on equilibrium concepts. For example, the modulation of dynamics was proposed to change the entropy of a protein, hence affecting processes such as binding. This so-called dynamic allostery scenario has been demonstrated in several recent experiments. Perhaps even more intriguing may be models that involve out-of-equilibrium operation, which by necessity require the input of energy. We discuss several recent experimental studies that expose such potential mechanisms for coupling dynamics and function. In Brownian ratchets, for example, directional motion is promoted by switching a protein between two free energy surfaces. An additional example involves the effect of microsecond domain-closure dynamics of an enzyme on its much slower chemical cycle. These observations lead us to propose a novel two-time-scale paradigm for the activity of protein machines: fast equilibrium fluctuations take place on the microsecond-millisecond time scale, while on a slower time scale, free energy is invested in order to push the system out of equilibrium and drive functional transitions. Motions on the two time scales affect each other and are essential for the overall function of these machines.

Multiple proteins operate as machines: They employ energy in order to perform a specific task in a cyclical manner. Protein machines can function as motors and carry loads, act as enzymes and perform chemical transformations or manipulate other macromolecules in order to drive various biological processes. However, the operation of protein machines is very different from that of machines familiar from our daily life. They need to work under a regime of constant collisions with solvent molecules, multiple small solutes and other macromolecules. Further, they may derive their energy from binding of some of these small solutes. Finally, their internal free-energy landscapes are rugged and therefore involve multiple stable configurations, which are termed “conformational states”. An outcome of these operational conditions/principles is that the functional cycles of protein machines are stochastic rather than deterministic, and involve random binding of substrate molecules, random transitions between functional states and random release of products. Understanding the principles of operation of protein machines and the involvement of such stochastic events in their function has been a goal in biophysics for many years; new experimental tools are shedding fresh light on this topic and providing novel ideas.

As noted above, the free-energy landscapes of proteins are rugged and complex (Figure 1A), which leads to a broad spectrum of internal motions, taking place on multiple time scales.^1,2^ These so-called “conformational dynamics” include local side chain motions on the femtosecond-picosecond time scale, motions of loops, helices and other secondary structure elements on the nanosecond-microsecond time scale, and large-scale fluctuations of domains and even subunits that can take place on the microsecond-millisecond time scale (Figure 1B). Are these motions just random, thermal fluctuations, or have some of them evolved to be important for the functional steps of a particular protein machine?

**Figure 1:**
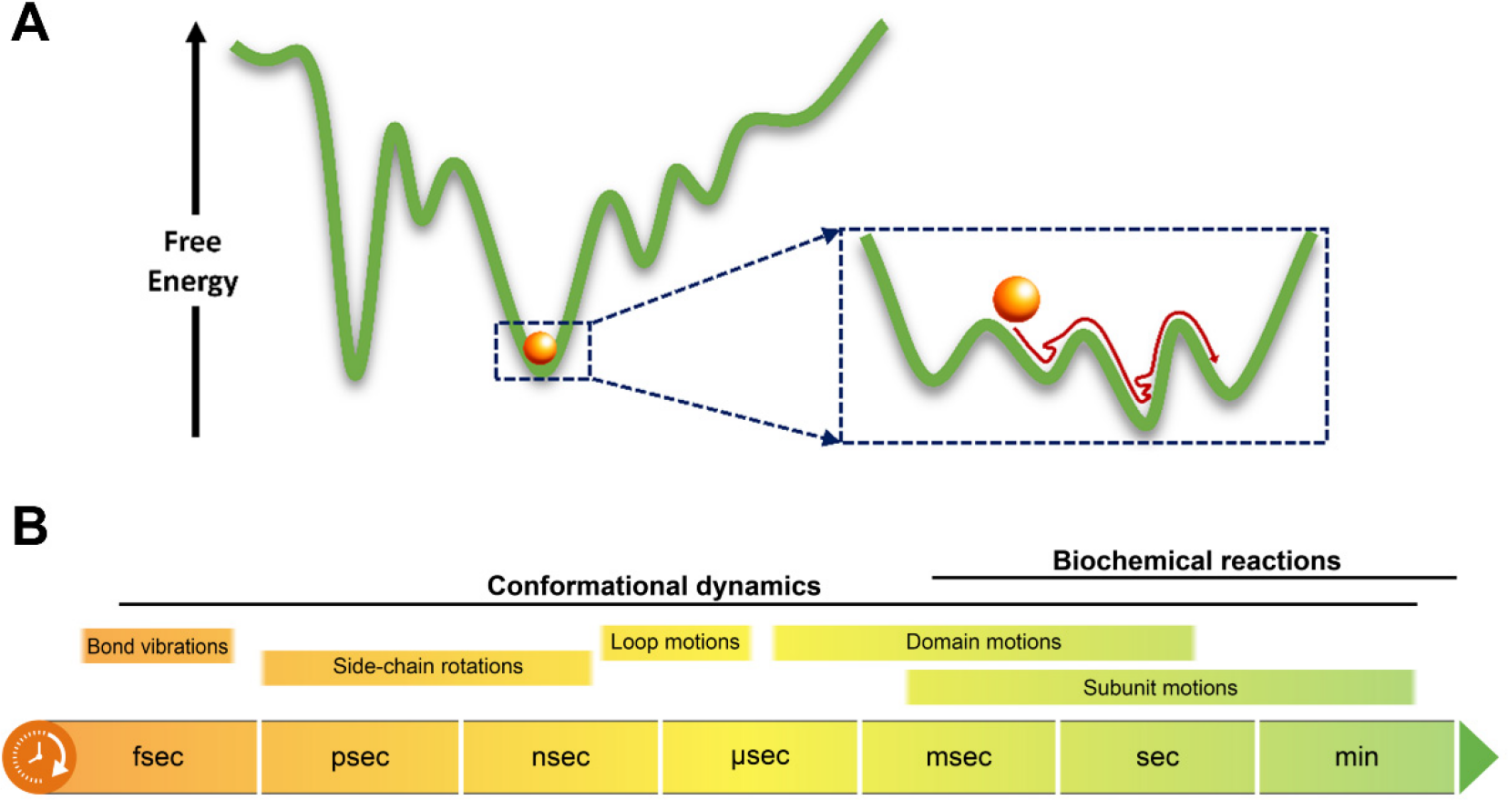
Proteins are dynamic. (A) The free-energy landscapes of proteins are rugged, with multiple free-energy minima separated by barriers of a broad spectrum of heights. (B) Time scales of protein dynamics, from the very local motions to global domain and subunit rearrangements. The biochemical reactions of proteins usually take place on longer time scales than typical conformational transitions.

The effect of conformational fluctuations on function is often related to the concept of “allostery”, which refers to the ability of proteins to transmit a signal generated by ligand binding at one site to a far-away site. This signal transmission is a means of functional regulation.^3,4^ In multisubunit proteins like hemoglobin, substrate binding to one subunit affects substrate binding to all other subunits. The allosteric effect leads in this case to a cooperative response. However, allosteric effects can also operate within single-subunit proteins and may involve different types of interactions, not necessarily all related to ligand binding. The traditional view of allosteric transitions links them to conformational changes between quasi-stable states on the free-energy landscape of a protein. Allosteric transitions involving conformational changes can be formalized in terms of familiar and often-reviewed models.^5,6^

As will be seen below, this is not the only way by which allostery operates. Intriguingly, functional steps of proteins, such as the chemical reaction of an enzyme or the spatial motion of a motor, are often much slower than internal dynamics and may take milliseconds to seconds and even longer. These functional steps are sometimes dictated by “external” events, such as the binding of substrate molecules or the release of products.

Therefore, a major question arising from the above is whether there is a connection between fluctuations and motions on fast time scales and the much-slower functional dynamics. This is the topic of this Perspective. We will discuss current thinking about the coupling between fast and slow transitions in proteins and introduce recent findings that point to and illustrate novel models, with some emphasis on results from our lab.

## Equilibrium Effects

If conformational dynamics are significantly faster than the functional steps of a protein machine, it is possible that the time scale of the dynamics is less of an issue and it is rather *the modulation of the free-energy landscape of the protein* (an equilibrium quantity) that contributes to protein function. Such equilibrium effects are discussed in this section.

### Dynamic Allostery

**Figure 2:**
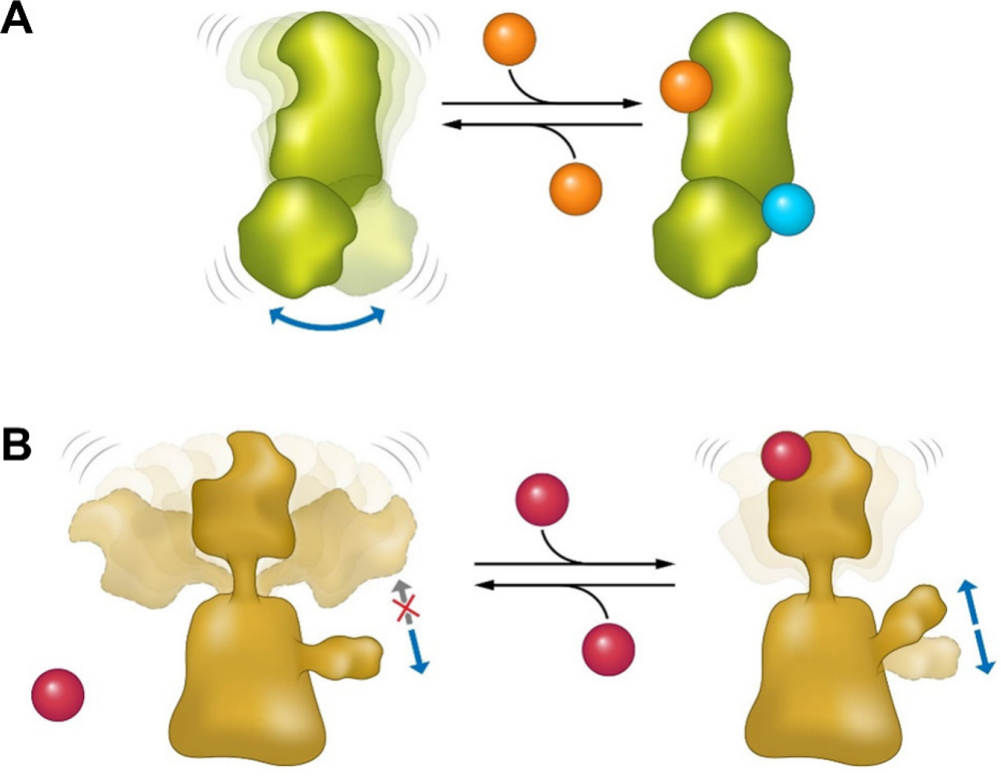
The diverse effects of protein dynamics on function. Equilibrium effects. (A) Dynamic allostery: The binding of a ligand to a protein changes its spectrum of motions, which serves as an allosteric signal to promote the binding of a second ligand. (B) Entropic inhibition: The motion of a protein domain limits the conformational space of another domain. Upon ligand binding, this motion is restricted, allowing the other domain to change its conformation.

In a seminal and prescient paper, published in 1984, Cooper and Dryden (CD) analyzed the statistical thermodynamics of ligand binding to a protein with two binding sites.^7^ They showed that by modifying the spectrum of fluctuations of a protein, ligand binding may lead to an overall change in the entropic part of the protein’s free energy, which can be translated into a modulation of the binding of another ligand at another site. In the CD model, the entropic change, which is due to modulation of dynamics, is large enough to explain cooperativity between two binding sites, even in the absence of conformational changes. The “dynamic” change induced by ligand binding may modulate the vibrational or conformational density of states of the protein. This is an equilibrium effect; the modulation caused by the binding of a ligand to one site on the protein involves internal motions occurring on multiple time scales. These motions are significantly faster than the functional time scale of the protein, and therefore a change to the free energy is incurred, which affects ligand binding at another site (Figure 2A). The CD proposal was surprising and very intriguing, and it took some time to demonstrate experimentally this so-called “dynamic allostery” effect. In 2006, Popovych et al. showed that binding of cAMP to one subunit of the dimeric catabolite activator protein affect motions in the second subunit, thereby changing the entropy of the protein.^8^ Since no conformational changes were observed upon binding, the allosteric effect could be attributed to the entropic contribution of the modulation of dynamics, just as proposed by CD. Several additional cases of dynamic allostery have been reported since then. For example, Petit et al. showed that either removal or phosophorylation of a specific α-helix of a PDZ domain leads to enhanced side-chain dynamics throughout the domain and concomitantly a significant reduction in its affinity to a peptide ligand.^9^ Capdevila et al. found that Zn binding to the transcriptional regulator CzrA redistributes fast local motions, which affects the binding of the protein to DNA.^10^ Hilser and co-workers argued that the concept of dynamic allostery can be generalized to encompass both local unfolding transitions and intrinsically disordered segments in proteins.^11^

### Generalizations of Dynamic Allostery

While the original dynamic allostery phenomenon involves changes in the rigidity of a protein, mostly modulating local motions, there might be further ways by which equilibrium entropic effects can contribute allosterically to protein function. Two such remarkable modes of dynamic allostery have been discovered in our recent studies of ClpB. This hexameric bacterial protein rescues protein molecules from aggregates.^12^ Each subunit of the disaggregation machine contains a coiled-coil structure, the middle domain (M domain), which is known to act as the activator of the machine. When the M domain’s conformation is perpendicular to the long axis of ClpB, it cannot bind the co-chaperone DnaK, and consequently machine activity is suppressed. When the M domain tilts, it binds the co-chaperone, and activity is enhanced.

Single-molecule FRET studies revealed that, rather than stably populating one of its two states, active and inactive, the M domain toggles between them on the submillisecond time-scale.^13^ This motion is several orders of magnitude faster than the overall activity cycle of the protein. This finding implies that on the slow time scale of disaggregation (seconds), the protein can only sense the population ratio of the two states of the switch, rather than its momentary state. Interestingly, this population ratio depends on external factors such as the binding of the co-chaperone DnaK. The M domain therefore becomes a tunable, analog switch, rather than the originally proposed two-state, digital toggle. The switch is found to turn on the disaggregation activity of the protein over a narrow range of values of its active/inactive state population ratio.^13^ This form of dynamic contribution to allostery can therefore be termed “molecular digital-to-analog conversion”.

Interestingly, microsecond motions of the N-terminal domain (NTD) of ClpB were observed to restrict the conformational space of the M domain in the absence of a substrate protein. In particular, the NTD visits a large population of conformations, some of which are sterically preventing the M domain from visiting part of its own conformational space. This restriction effectively prevents the M domain from tilting and thereby activating ClpB.^14^ The binding of a substrate to the NTD prevents it from occupying its whole range of conformations, thereby removing the motional restriction and enabling activation of the machine through the tilting of the M domain. Since the effect involves changes in the conformational space of the NTD, we called it “entropic inhibition” (Figure 2B).

## Dynamic Effects

So far, we discussed situations where conformational dynamics contribute to function by modulating the equilibrium distribution of states. In other words, and as already pointed out, the models and examples introduced in the previous section dealt with changes to the free energy of a protein mediated by entropic effects. The question arises whether there are cases where the effect of dynamics goes beyond equilibrium. In such cases, the dynamics truly affect the outcome of a particular protein function by influencing *the time course of the reaction*. Dynamic effects must operate out of equilibrium, which simply means that the relative populations of conformational states of the protein are different from those expected under equilibrium conditions. A constant input of energy is required in order to maintain a system in an out-of-equilibrium state. It is important to note that, given that both conformational dynamics and functional transitions in a protein are stochastic, it is not expected that a direct link exists between conformational and chemical degrees of freedom. Indeed, a direct link would require some form of synchronization that is very difficult to achieve, given the disparity of time scales of the fluctuations involved. This comment pertains to proposals that so-called “promoting vibrations” may operate in proteins; for a detailed discussion of the difficulties with these proposals, we refer the reader to a Perspective written by Warshel and co-workers.^15^

**Figure 3:**
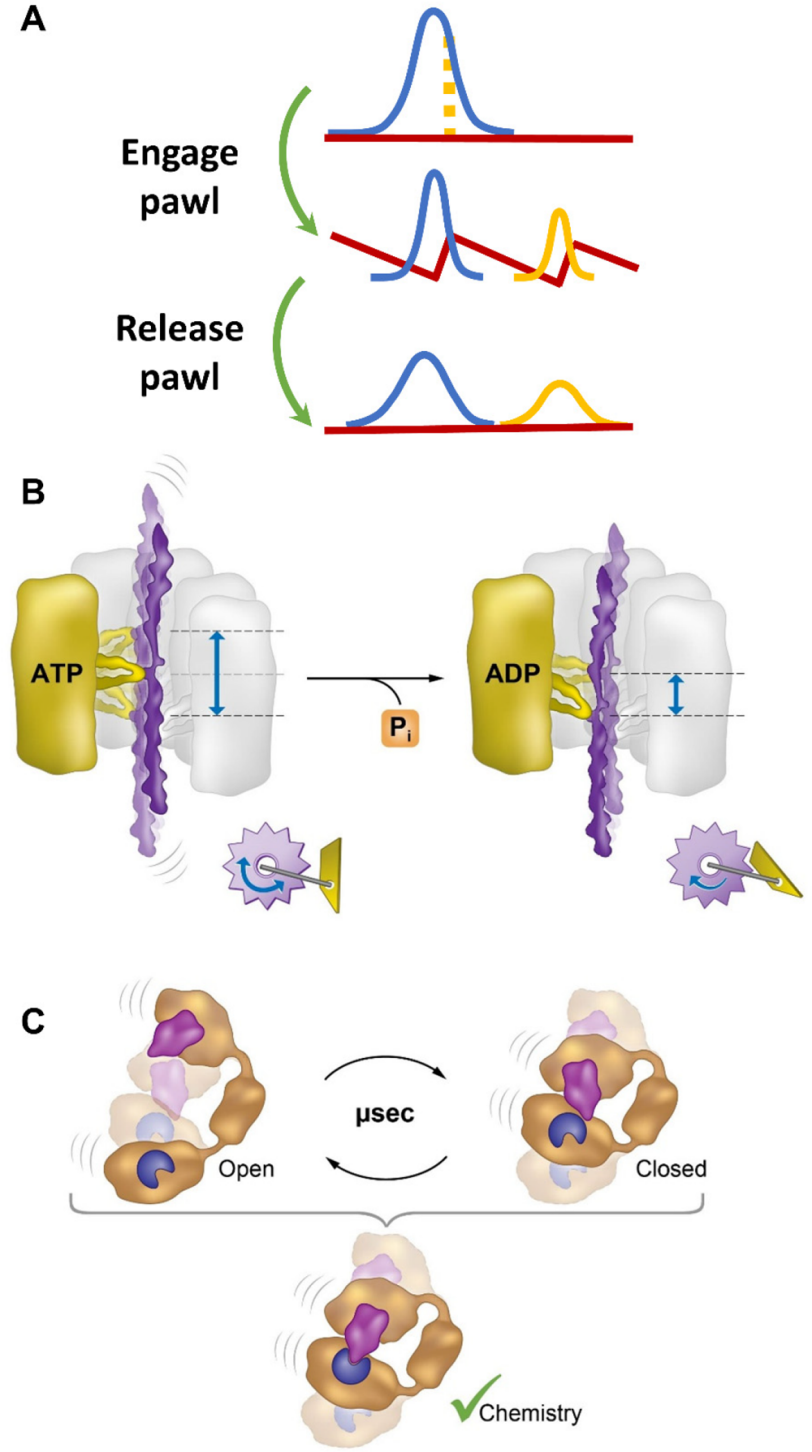
The diverse effects of protein dynamics on function. Dynamic effects. (A) In a simple version of a Brownian ratchet, an effective pawl periodically switches the molecular dynamics between a flat free-energy surface and a structured surface, promoting unidirectional motion. Adapted with permission from ref (16). Copyright 2021 AAAS. (B) Pore loops in the lumen of a protein machine may serve as Brownian ratchets, using an energy source (ATP hydrolysis) to transit between two free-energy surfaces, with one of them potentially more restricted than the other, similar to the states of a macroscopic ratchet. Motion of a substrate protein in both directions is possible before ATP hydrolysis, which is equivalent to a ratchet with an unengaged pawl. Following ATP hydrolysis, the pawl is effectively engaged and the motion is rectified. Insets demonstrate a macroscopic ratchet in its two states. (C) Optimization of enzymatic substrate binding by fast dynamics: When substrates are bound in the wrong configuration for the enzymatic reaction, fast domain motions may allow them to search for the optimal configuration and react.

In this section, we will discuss what we view as bona fide dynamic effects on function. We will introduce here the Brownian ratchet (BR) mechanism (Figure 3A) and provide examples where it has been implicated in protein activity. Recently, work from our lab has directly measured conformational dynamics that can be related to a BR mechanism,^16^ as will be discussed in some detail. A second and different mechanism for the coupling of fast and slow transitions will also be considered, namely the annealing of protein-bound substrate conformations by fast conformational changes in preparation for a much slower biochemical reaction.^17^ Combined experiments and simulations have pointed to the potential importance of this mechanism for enzymatic activity.

### Brownian Ratchets

A rigorous mechanism for dynamically coupling fast and slow time scales is provided by the BR concept.^18^ In a macroscopic ratchet, the engagement of a pawl generates unidirectional motion. In the generic Brownian version of such a device (Figure 3A), the motion of a particle is alternated intermittently between two free-energy surfaces.^18^ The first free-energy surface is structured such that fast Brownian motion is unhindered. The structure of the second free-energy surface, on the other hand, restricts the motion, e.g., by introducing a particular pattern of free-energy barriers. Alternation between the two free-energy surfaces may rectify the motion, in a seeming defiance of the expectations of equilibrium thermodynamics. In reality, there is no violation of any physical rule, as a constant investment of energy is necessary for the slow switching between the two free-energy surfaces, making this an out-of-equilibrium reaction. In the biological setting, the switching between free-energy surfaces is typically driven by a biochemical reaction, such as ATP hydrolysis, and the specific type of ratchet arising in this context has been called an “information ratchet”.^19^ Brownian ratchets of different types have been analyzed in detail in the physical literature, and the discussion of questions such as the optimal parameters to efficiently generate directional motion can be found in reviews such as refs (18 and 20).

A very well-characterized example of a BR is provided by the Na,K-ATPase, a gated ion pump. Impressively, it has been shown that an external alternating electric field can drive the pump even in the absence of ATP, and it has been suggested that this is due to conformational switching similar to the one occurring in the ATP-driven cycle of the protein.^19^ There is some debate whether other molecular machines operate with a BR mechanism or a power-stroke mechanism. The latter, which is more akin to the function of macroscopic machines, involves an abrupt conformational jump following an energy-consumption step^21^ and does not require operation on two time scales, as in a BR. Recent work suggests that power strokes can be viewed as design elements within more generalized BR models.^22^

Our work on the disaggregase ClpB has led us to propose a BR mechanism for its protein translocation activity.^23^ Substrate proteins threaded by ClpB are engaged by pore loops, structural elements protruding into the central channel of the protein.^12,24^ Each subunit of ClpB consists of two nucleotide-binding domains (NBDs); NBD1 contains two pore loops, PL1 and PL2, while NBD2 contains a single pore loops, PL3. The hand-over-hand mechanism proposed for substrate translocation by AAA+ machines, based on structural data, assigns a static role of substrate gripping to the pore loops.^24^ In contrast, single-molecule FRET spectroscopy^16^ identified microsecond motions with significant amplitudes in all three pore loops. It was found that the dynamics of each pore loop could be represented by two states, either “up” or “down”. In the case of PL2 and PL3, the populations of the two states were different in the presence of ATP, ADP or ATPγS (a slowly hydrolyzable version of ATP). In contrast, the two states of PL1 were not sensitive to the nucleotide identity, implying that PL1 is ATP-hydrolysis independent. Mutagenesis of key residues in PL1 and PL3 identified a correlation between their dynamics and the disaggregation activity. Taken together, these results led to a suggestion that the pore loops of ClpB operate through a BR mechanism.^16^ In particular, PL1 and PL3 seem to both contribute to pulling substrate proteins through the central channel of ClpB. Further, since PL1 is ATP-hydrolysis independent, it is likely to engage and pull substrate proteins without being affected by the nucleotide status of the machine. On the other hand, the hydrolysis-dependent PL2 and PL3 may act as pawls to rectify substrate pulling, thereby avoiding slippage and enabling directional motion (Figure 3B). Importantly, in AAA+ machines with a single NBD, like ClpX, only a single pore loop exists in each subunit,^25^ and it therefore needs to act as both a puller and a pawl. This pore loop is the equivalent of PL3 in ClpB, which explains why the latter has a dual role. To recap, a BR may act within ClpB by harnessing the motions of its pore loops. Fast fluctuations of the pore loops can move substrate proteins through the central channel of ClpB. Slower, energy-driven changes originating at the ATP-binding sites may allow some of the pore loops to rectify substrate motion and prevent slippage and release from the wrong direction.

The BR mechanism proposed for ClpB is commensurate with the findings of very fast protein translocation in optical tweezers experiments on ClpB,^26^ as well with mutational studies that identified the ability of the protein to thread substrates even if not all subunits are operational.^27^ It remains to be determined whether the BR operates in parallel to the hand-over-hand mechanism or rather replaces it.

### Fast Fluctuations Promote Substrate Rearrangement

It is often proposed that substrates bind to enzymes at a well-defined conformation, as their appropriate orientation is crucial for the catalytic reaction.^15^ This is likely correct when an enzyme’s active site is prestructured and relatively rigid. However, when an enzyme binds two substrates, as is quite often the case, it is less likely that both substrates will bind rigidly to form a configuration that is ready for the chemical step. More often, the enzyme needs to undergo a conformational transition that will bring the two substrates close together. Such a structural change requires some flexibility in the enzyme. What then guarantees that the initial configuration of the substrates when they bind to the enzyme will be the correct configuration for the reaction to proceed?

The enzymatic reaction of the relatively small enzyme AK is a good example for the above scenario; it requires a domain-closure conformational change in order to bring ATP and AMP close together for a phosphoryl transfer reaction. It has been shown that the phosphoryl transfer step requires an accurate relative positioning of the two substrates.^28^ Early studies, using both NMR and single-molecule spectroscopy, suggested that domain motions, particularly domain opening, are rate limiting for the enzymatic reaction of AK.^29,30^ However, recent work demonstrated that the rates of domain closing and opening in AK are much larger than the turnover rate of the enzyme, which is ∼400 s^–1^.^17^ Surprisingly, the rate of domain closure in AK molecules bound with substrates was measured to be as high as 65,000 s^–1^, with a domain opening rate of ∼20,000 s^–1^.

To explain how domain motions in AK that are so much faster than the enzymatic turnover may still contribute to the function of the protein, we resorted to the concept of “bath fluctuations”, which is familiar in chemical physics. Bath fluctuations are the stochastic motions of those degrees of freedom that are not involved in a reaction. For example, in electron transfer reactions, fluctuations of the solvent can bring the electron donor and acceptor to a state in which their energies are equal, in which case an electron can readily jump from one to the other.^31^

Is it possible that fast domain motions of AK serve a similar role and bring the two substrates to a region on the free-energy landscape that is conducive for the reaction? The fast opening and closing of the domains may gradually “anneal” the substrates, which can bind initially in an incorrect orientation, and allow them to reach the correct one (Figure 3C). This model was tested recently in a series of nonequilibrium molecular dynamics simulations,^32^ which started from a configuration where ATP was bound correctly to the enzyme, but AMP was bound incorrectly. Following several cycles of domain closing and opening, the AMP molecule reached the correct configuration for the reaction. The simulations therefore provided a strong support for the conceptual picture introduced above and placed it on a solid ground: Fast domain motions have an effect on a much slower enzymatic reaction through their modulation of substrate conformation. The nonequilibrium aspect of this mechanism involves the initial incorrect binding of the substrates and the gain of free energy arrived at through their annealing to the correct configurations.

Large-scale conformational dynamics that are significantly faster than function were also reported for other enzymes, even if the context introduced above, i.e., optimization of substrate conformation, had not been necessarily discussed. We will mention here studies of T4 lysozyme,^33^ the electron-transfer enzyme quiescin sulfhydryl oxidase^34^ and the influenza A RNA polymerase PB2.^35^ These studies demonstrated microsecond and millisecond jumps between conformational states of the proteins. Such conformational dynamics could well be of similar importance to the substrate conformation-optimizing dynamics of AK.

## Conclusions

There is no doubt that dynamics play an important role in the function of proteins. Proteins fluctuate on multiple time scales, and these fluctuations contribute to the entropic part of their free energy. As discussed above, the contributions of fluctuations to the free energy of a protein may affect specific processes such as allosteric transitions, even in the absence of changes to average conformations. Moreover, and quite obviously, fluctuations contribute to any process that requires a free-energy barrier crossing, such as an enzymatic reaction. However, a question that has intrigued biophysicists for a long time is *whether dynamics can contribute to protein function beyond thermodynamic equilibrium*. Such a contribution must involve out-of-equilibrium scenarios.

We described in this Perspective several cases in which a bona fide involvement of dynamics is plausible. Remarkably, these cases typically involve both fast and slow processes. Models in this class can be unified under what we would like to call *the two-time-scale paradigm* for biological machines: Fast fluctuations take place on the microsecond-millisecond time scale, while on a slower time scale free energy is invested in order to push the system out of equilibrium. The interaction between motions on the two time scales is necessary for the function of these machines. Given the separation of time scales, the fluctuations on the fast time scale bring the system into a quasi-equilibrium state. The familiar example of the BR model, which seems to be relevant for multiple biological machines, involves slow and energy-consuming switching between (at least) two free-energy surfaces, on each of which much faster motions can occur. Perhaps a bit less familiar is the case of substrate conformation optimization following binding to a protein, as in the case of AK. Here, the out-of-equilibrium component can be attributed to the binding event itself, and the system eventually decays to equilibrium before a chemical step takes place.

The generality of *the two-time-scale paradigm* remains to be established as additional cases of conformational dynamics coupled to function are analyzed by advanced experimental and computational tools.^36^ It is also likely that other mechanisms by which conformational dynamics contribute to protein function will be unearthed in such studies. Further, there are proteins that have been optimized to perform their function without the need to undergo significant internal motions, such as carbonic anhydrase, some variants of which turnover product at the astounding rate of a million times per second.^37^ Such an extreme structural optimization can happen only in specialized cases, and most enzymes and protein machines are likely to require significant internal motions for their function. Given that the typical times for large-scale motions within proteins are expected, based on fundamental arguments,^38^ to be rather short, it would not be surprising if a picture similar to the above emerges in studies of multiple proteins.
